# Microstructures and dynamic processes within the five-phase system: regarding COVID-19 as a complex system

**DOI:** 10.1186/s11782-020-00090-6

**Published:** 2020-09-16

**Authors:** Fengbin Wang, Xue Zhang

**Affiliations:** 1 Business School, Renmin University of China, Beijing, 100872 China; 2 Center for Management Philosophy and Organizational Ecosystem, Renmin University of China, Beijing, 100872 China

**Keywords:** Five-phase system, Coronavirus disease (COVID-19), Balancing feedback, Microstructure, Spiraling process

## Abstract

The research uses the development of COVID-19 in the human body as an example to explore the microstructures and dynamic processes of a concise complex system from the lens of the five-phase system. Based on the structural balance theory and system dynamics, the research finds that transitive triads and cyclic triads in the five-phase system are both imbalanced. The integration of these differentiated triads comprises of a balanced intermediate form in the shape of quadrangular cycles. These cycles serve as microstructures of the five-phase system, due to the inherent balancing feedback mechanism, and support the generation of resultants. The alternation of quadrangular cycles drives the spiraling development of the whole system. By orderly and regular interweaving of signed directed links, the research provides a holistic, process-oriented demonstration for the development processes of COVID-19. It clarifies that the essence of the five-phase system is phase-transition processes with the quadrangular cycle as carrier and supporter, rather than the static aggregation of five elements. The research deepens the understanding of system nonlinearity by visualizing the circular causality and promotes the academic dialogue between the Western process theory and the Chinese inherited notion of the five-phase system.

## Introduction

The severity and prevalence of the coronavirus disease (COVID-19) worldwide have prompted medical experts to conduct an increasing number of studies on the progression of the novel coronavirus (2019-nCoV) in the human body. From the perspective of general management, the development of 2019-nCoV in the human body could be regarded as a complex system in which interactive elements such as the coronavirus, symptoms, and the immunity system form a networking structure. Thus, understanding the structure and its operation pattern through the domain-interaction inquiry (Cornelissen 2005) across both health management and organizational management would provide new insights to deal with the sudden burst of COVID-19 from the perspectives of management and organization study. The five-phase (*wuxing*, 五行) system originated from ancient China, the combination of structural balance theory and system dynamics illuminate the exploration of the microstructures and dynamics of the complex system.

Shifting focus from entities to relations and moving the called-upon systems thinking (Gharajedaghi 2011) beyond compositional complication to relational complexity, the notion of five-phase system inherited from ancient China regards any entity as a dynamic and interconnected system. It appears in sharp contrast to both atomic and static thinking in the existing mainstream studies. Applying the notion of the five-phase system to the process study on complex systems that are in flux and transformation (Morgan 2006) would help to extricate the relationships among different elements and enrich the knowledge of phase transitions that make the elements change their qualities in succession. Through this new perception, the trajectory of emergence would be unfolded, which may derive from the microstructures of the focal system. Currently, the internal structures of a complex system are examined from the lens of structural balance theory and system dynamics, the comparison, and the combination of which implies that the fundamental structure of the five-phase system may be triads. However, the triads’ nature of closure (transitive or intransitive), structural property (balance or imbalance) and the specific pattern of their structural integration have not been clearly and consistently identified. These underlying confusions require a review of different kinds of triads and their balancing property, which may be done by the interweaving of positive and negative directed ties of the five-phase system. This research enhances the understanding of the focal system that is in flux and transformation, and simultaneously promotes the mining of modern values of the ancient five-phase system and academic dialogue between Eastern and Western theories.

Consistent with the paradigm shifting from the atomistic account of being to the processual account of becoming, this research intends to uncover the dynamics and implicational law underlying the endogenous evolution of complex systems. The logic of analogical abduction (Mantere and Ketokivi 2013; Peirce 1878) is adopted to use the development of COVID-19 in the human body as a metaphor for providing a plausible explanation on the evolution of system states resulting from the internal tensions between opposites.

Regarding the development process of 2019-nCoV in the human body as a complex system, the research has found that the intensity of 2019-nCoV, symptoms of the disease, organ changes, immunity reaction, and physical condition together constitute a five-phase system. In this, transitive triads and cyclic triads perform different functions and are detected as microstructures of the five-phase system. As the integration of these two kinds of triads, a novel structure shaped like quadrangular cycles is constituted, which turns out to be balanced meso-level structures containing reactant, mediator, resultant, and catalyst, and functionally supports the generation of resultant. When the first quadrangular cycle ends, the catalyst turns into the reactant and activates the second quadrangular cycle. This is isomorphic with the first one. The successive two quadrangular cycles form a macrostructure in the shape of a pentagon. The alternation and circulation of several quadrangular cycles promote the spiraling development of the five-phase system. This uncovers the endogenous dynamics of system emergence and evolution.

The research explores the microstructures and phase-transition processes of COVID-19 as an analogy of the evolving complex system. It deepens the understanding of the holistic and dynamic phase transitions of complex systems through the practice of domain-interaction inquiry. There are several potential contributions to the research of complex systems in general and organizations in particular. Firstly, the research contributes to the merger of structural balance theory, system dynamics, and the five-phase system. Secondly, it extends the understanding of system nonlinearity by demonstrating circular causality. Thirdly, it reveals the phase-transition processes with quadrangular cycles as building blocks of the five-phase system, deepening the knowledge of dynamic and holistic features of complex systems. Lastly, the research visualizes the characteristics of pragmatic philosophy, enriching the philosophical connotation of the five-phase system.

## Literature review

### Five-phase system and pragmatism philosophy

Proposed by the ancient Chinese philosophers, especially the Daoist, the notion of the five-phase system has been increasingly acknowledged as a correlative cosmology (Law and Kesti 2014) valuable to unveil the never-ending cyclical processes underlying the operation of everything in the universe. This philosophical notion has been applied in the practices of traditional Chinese medicine (TCM) (frequently with each *xing* associated with metal, wood, water, fire, and earth, respectively) and occasionally but growingly used in the holistic and dynamic pattern of explanation on the phase transitions in natural, social and political phenomena. Considering the development and influences of COVID-19 that are affected by both external and internal forces, we regard that the notion of five-phase system could provide deep insights into the relationships of human bodies with the environment. Thus, it is beneficial to promote scientific articulation on the micro-foundations and dynamic processes of COVID-19 as a kind of complex phenomenon.

Different from the mainstream Western atomic thinking on separated entities, the philosophical notion of the five-phase system stresses the holism and dynamic balance and has gradually accomplished as the theoretical core of relational thinking (Chen and Miller 2011; Langley and Tsoukas 2016a; Nayak 2008; Shipilov et al. 2014). Although inherited from ancient China, this notion suits the pragmatism philosophy that advocates anti-dualism thinking. As Hartshorne (1998) states, it is a misconception to distinguish as if there is only either becoming or being. Understanding the nature of complex reality demands a holistic and dynamic way of seeing that looks beyond the becoming–being distinction (Schoeneborn et al. 2016) and adopts a strong process view to conceive entities as temporary instantiations of ongoing processes (Burgelman et al. 2018). Morgan’s (2006) metaphor that images organizations as flux and transformation has signified a paradoxical relation between being (an entity) and becoming (a process) (Cornelissen 2005). However, the majority of process studies focus on how processes produce organizations/structures. In contrast, the adverse causation as how (micro) structures shape the processes of emergence is seldom inspected, which invites the combination of both, such as the call of “recursive structuration” (Kouamé and Langley 2018; Wu and Sekiguchi 2019).

Process philosophers opine that being and becoming are entwined (Helin et al. 2014; Hernes 2016; Tsoukas and Chia 2002). For highlighting the self-production of being in becoming (Langley and Tsoukas 2016b), pragmatist pioneers, including Henri Bergson (1859–1941), Alfred North Whitehead (1861–1947), and Martin Heidegger (1889–1976), have engaged intensely with the temporal process of “creative evolution,” or “making present,” to inquire and account the way that “from nothing, something emerges, and other things fall away into nothing” (Helin et al. 2014). Since how something becomes is always a question of how it is related and what forces are at play in such relations, the processual orientation awakens an ontological account of enfolded relationships in the structure of parts that constitute an evolving whole. This simultaneous account of the parts and whole, as well as the structure and process, are congruent with the Daoist notion of “coming together of opposites,” i.e., the dynamic interplay of *yin* and *yang* (Morgan 2006).

As Zhang (2017) stated, events unfold utilizing the interplay of opposition and simultaneous association and can only be understood by understanding the correlations between them. In the five-phase system, there are enabling links (→) between adjacent elements in the nearest, illustrated as wood creates fire, fire creates earth, earth creates metal, metal creates water, and water creates wood; meanwhile, for the illustration of constraining links (> −− ), wood is shaped by metal, metal is melted by fire, fire is extinguished by water, water is controlled by the earth, and the earth is broken by wood. The interpenetration of enabling and constraining relationships, thus, equips the formed five-phase system with an inherent capacity to achieve the dynamic balance deriving from the unity of opposites.

**Fig. 1:**
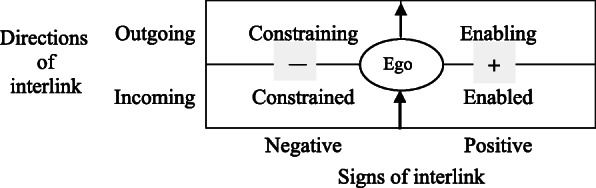
The bi-dimensional classification of interlinks centered on a designated ego

The five-phase system could be portrayed as a clear framework of a network-typed graph (shown in Fig. 1) restated from the perspective of social network theory to demonstrate the development process of a system in flux. The framework is only constituted by five-nodal components (thus, it is parsimonious or simple) that link with each other via signed and directed ties (thus, it is nonlinear or complex), thus forming a *simplex*^1^ structure. The Chinese phrase *wuxing* is frequently incorrectly translated into English as “five elements” (Law and Kesti 2014; Major 1976), while it is better to be interpreted as “five processes” (Needham 1954), “five modal phases” (Bennett 1978), “five phases” (Qu and Garve 2008) or “five evolutive phases” (Major 1976).

As shown in Fig. 1, combining both the signs and directions of interlinks centered on the ego into a bi-dimensional framework is meaningful. Together, the distinctions between positive and negative relations (horizontal ordinate), and that between active (outgoing) and passive (incoming) relations (vertical ordinate), present a typical style in Western literature. This fits well with the Chinese notion of the five-phase system, only if the five components of a system are redefined as nodes that are interactive with the ego through interlinks of constraining, enabling, enabled, and constrained, respectively.

Hypothetically, Fig. 1 is a signed and directed graph constituted by five types of nodes, including the constrainer, the enabler, the ego, the enablee (i.e., who is enabled by the ego), and the constrainee (i.e., who is constrained by the ego). Stated metaphorically, when a specified node, such as wood, is taken as the ego, the interactive nodes at a distance of two-step before/after it, metal and earth, would act as the ego’s constrainer/constrainee, and the nodes one-step before/after it, water and fire, would act as its enabler/enablee. All the four nodes related to the ego are contained in an integrated model, patterned as a pentagonal graph.

The five-phase system proposed by the ancient Chinese philosophers is a more dynamic model whose dynamics are thought of intrinsic, inside a living system. This notion is linked to, but goes beyond the identification of the five interactive nodes, using the term *xing* (movement) to highlight the relationships among the five dynamic processes.

### The judgment on balance and imbalance of triadic structures

Living systems are scarcely isolated but rather are interlinked spatially and temporally. However, most empirical studies of social networks focus on node properties while being blind to the multiplicity and/or dualities of the links connecting nodes (Szell et al. 2010). Therefore, Shipilov et al. (2014) highlight relational pluralism and temporal multiplicity, particularly pairwise relationships between two nodes. Besides, one main branch of social networks research, structural balance theory tends to focus on the small-sized human groups (Davis and Leinhardt 1967; Doreian and Mrvar 1996; Hummon and Doreian 2003; Sytch and Tatarynowicz 2014), leaving large-scale systems that usually contain non-human nodes under the concern of complex systems theory. But a notable inconsistency lies in the two disciplines, which urges us to differentiate their properties before attempting to achieve cross-disciplinary integration.

Recent researchers in network closures and dynamic processes endeavor to fill the research gap through reconfiguring the traditionally “unsigned” size-three groups as “signed” triads, which are small and simple in forms but exhibit complexity and dynamics due to the multiple relations inside the networks shaped by both positive and negative relations (Doreian and Krackhardt 2001; Leskovec et al. 2010). By clarifying the directions of ties in triads, Chase (1980) identifies two kinds of triads: transitive and intransitive triads. Transitive triad has a double attack (outgoing links) or double receive (incoming links) (e.g., A → B → C, A → C), and in an intransitive triad, the three ties form a closed cycle (e.g., A → B → C → A). Integrating the signs and directions of ties in triads, Huitsing et al. (2012) identify the typology of transitive closure and cyclic closure, which are similar to the classification of transitive triads and intransitive triads. Particularly, through the conceptual clarification and differentiation of complication (regarding the number of nodes) and complexity (regarding the nonlinearity of interlinks), the view of complex systems has been gradually connected with that of social networks. Thus, they attract scholarly attention on the sign of (undirected frequently) relations between and among interactive nodes, as in Table 1.

**Table 1:**
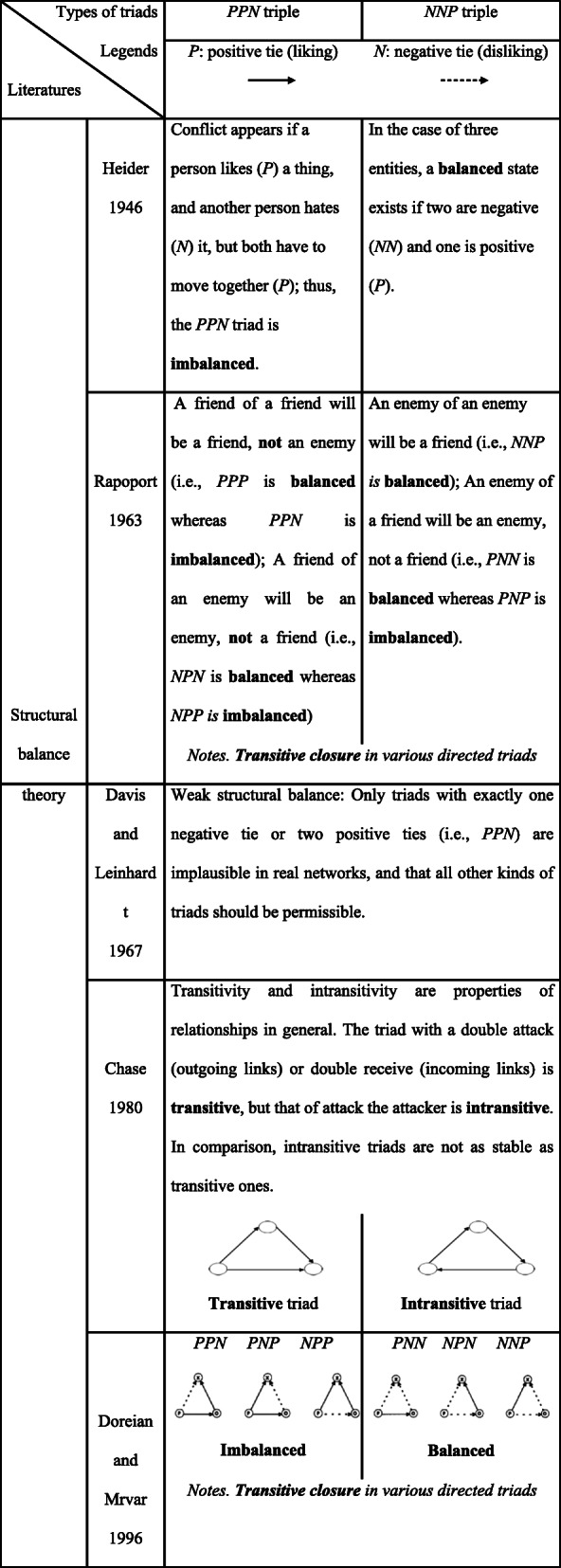

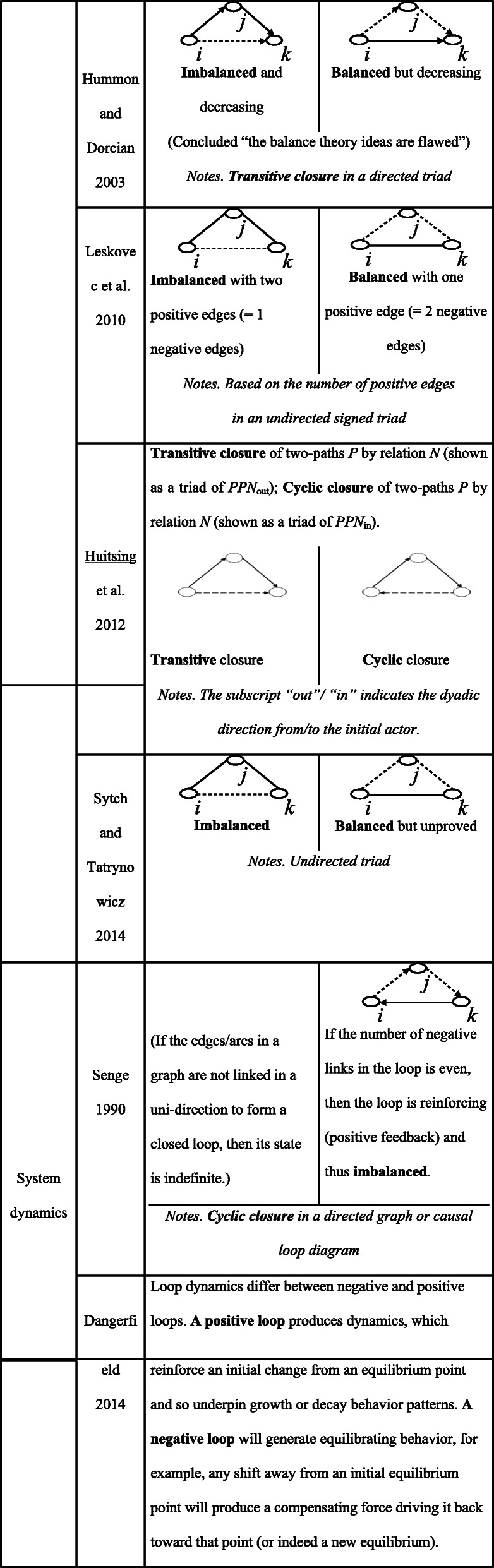
Balance and imbalance of two typical signed triads in the literature reviewed

As an essential part of signed network research, triadic closure is a fundamental step to unfold the process of network evolution (Zhelyazkov 2017). Still, its effects on system stability and sustainability are undetermined in the existing literature. Two firms collaborating with the same third party are unlikely to develop a conflictual relationship. Similarly, the triad composed of two positive ties and a new negative tie, shortly as *PPN* (*P* represents a positive tie, and *N* represents a negative tie), is considered out of equilibrium. Comparably, a “balanced” triad is analogical to Rapoport’s (1963) aphorism, “a friend of a friend is a friend” (*PPP*). That is, from the perspective of fundamental structural balance hypothesis (FSBH), a triad is considered as balanced if the product of the signs in the triad is positive and is imbalanced if the product of the signs is negative.

In the business or social reality, networks are not only complicated, containing more than three nodes, but also complex, exhibiting the coexistence of cooperative and competitive or co-opetition relations. Thus, a departure from FSBH, system dynamics theory, is regarded as an alternative to a macro-level investigation on the structure and functions of closed-looped systems. Accordingly, it is inferred that the odd-number of negative relations would imply a state of equilibrium, whereas the even-number of negative relations produces a non-equilibrium structure (Dangerfield 2014; Senge 1990). From the system dynamics perspective, we argue that both *NNP* and *NPN* are dynamic imbalanced. However, this argument is sharply contradictory to the FSBH’s typology (see the bottom part of Table 1). The inconsistency underlying the qualitative (yes or not) judgment of the structural balance of signed networks creates a puzzle that needs to be solved based on an in-depth investigation of different types of closure processes.

Huitsing et al. (2012) conduct empirical research on signed networks and identify two kinds of triad closure processes, that is, transitive and cyclic closures. Different from prior research, their research allows the induced relation of *i* with *k* to be directed either inwardly (*i* → *k*) or outwardly (*i* ← *k*) given the pre-closure conditions of two positive paths. Then, if the post-closure condition is a negative tie, the two positive ties closed by an incoming negative relation could make a *transitive* triad, which is imbalanced for signed networks according to the structural balance theory (Doreian and Krackhardt 2001; Heider 1946). In contrast, two positive ties closed by an outgoing negative relation could make a *cyclic* triad, which is balanced due to the inherent negative-feedback process based on the theory of system dynamics (Senge 1990).

In sum, the comparison of two disciplines of literature concerning triads (see Table 1) indicates the within-disciplinary consistent classification of *PPN* as imbalanced, whereas *NNP* as balanced from the lens of FSBH. Based on the number of positive edges in undirected signed triads, Leskovec et al. (2010) label triads with an even number of positive edges as imbalanced (such as *PPN*) and triads with an odd number of positive edges as balanced (such as *NNP*). For any undirected triads, by converting the number of positive edges into the number of negative edges, we identified that the state of imbalance depends on the number of negative edges being odd. The state of balance relies on the number of negative edges being even. This stream of inquiry on triadic closure is widely shared among the researchers in the structural balance theory. Still, because of the ignorance of interlink directions, its conclusion on whether signed triads are balanced is either indecisive (Doreian and Krackhardt 2001; Heider 1946) or in contradiction with the judgment via the lens of complex systems theory. The latter posits that the *NNP* triad is imbalanced, since its number of negative arcs (directed edges) is even, which implies a causal loop of reinforcing (positive feedback).

### A holistic analysis of human health and traditional Chinese medicine (TCM)

**Fig. 2:**
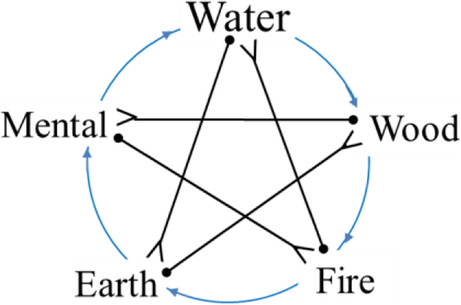
The model of five-phase system

In the past decade, TCM has gained increasing attention worldwide. Inheriting the traditional Chinese culture that emphasizes the nature of change, TCM defines health in terms of dynamics and harmony. Based on the classic perspectives of *yin* and *yang* (Li 2012, 2014, 2016) and the five-phase system, TCM is regarded as a clinical practice complying with “natural rules” of cosmos and has stood the test of more than two thousand years history. The five-phase system containing wood, fire, earth, metal, and water, is used initially to explain the composition and phenomena of the physical universe, including human bodies. The human body, regarded as a microcosm in the universe, has affinities in nature with the five components of the living organism. Chinese medicine likens the lung, liver, kidney, heart, and spleen in the human body to metal, wood, water, fire, and earth, respectively. The positive and negative links within a five-phase system (as shown in Fig. 2) are endowed to describe the mutual pathological influence and transmission among the viscera. For instance, one disharmonious organ may be affected by another organ, and meanwhile, it may affect some other organs.

TCM holds a holistic view of the human body and health. It stresses the overall, delicate harmony and coordination among different parts of the human body (Dong and Zhang 2001). It greatly emphasizes the careful readjustment and maintenance of this natural balance in the body. Health implies harmonious coordination among the various parts of the body and their adaptation to the natural environment. The fundamental mechanism of a disease is the breakdown of relative equilibrium within the organism or between the organism and its environment.

Based on the TCM, whether COVID-19 can be cured, as an intractable problem, would depend on the outcome of the struggle between the vital energy of the human body and the pathogenic factor 2019-nCoV, which attacks the person. TCM emphasizes the maintenance and transference of the vital energy and therefore is favorable at preventing the occurrence of disease and its further development.

Mainly composed of herbs, TCM could offer natural, safe, efficient therapies and cures for many diseases with much fewer side-effects than the antibiotics. In the prevention and treatment of COVID-19, TCM has been broadly adopted, especially in treating cases of mild symptoms (Ren et al. 2020). *Lianhuaqingwen* (连花清瘟), a Chinese patent medicine composed of 13 herbs, has played a positive role in the treatment of COVID-19. Recent research conducted by Zhong Nanshan and his academic team shows that *Lianhuaqingwen* significantly inhibits the 2019-nCoV replication, affects virus morphology, and exerts anti-inflammatory activity in vitro, making its use a novel strategy for controlling the COVID-19 disease (Li et al. 2020).

## A process study of COVID-19 development

### The development of COVID-19 in the human body from the lens of the five-phase system

**Fig. 3:**
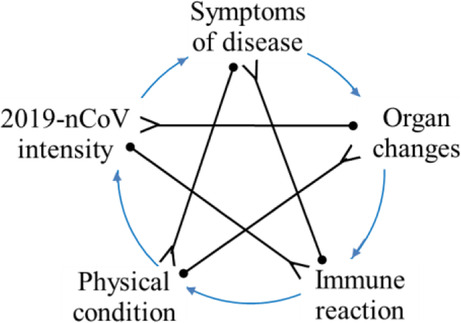
The system of COVID-19 development

As an essential constituent of Chinese scientific holism, the notion of the five-phase system provides prominent insights for explaining the phases of COVID-19 development in humans infected by 2019-nCoV (as shown in Fig. 3). When 2019-nCoV invades a human, it causes symptoms such as fever and dry cough. These symptoms lead the infected person into a state of gradual change of his/her organs. Simultaneously, the 2019-nCoV attacks the infected person’s lungs and other organs directly, resulting in physical and functional damages when it reaches sufficient concentration. In response, the attacked organs instruct that the body activates the immune system to fight the coronavirus by mobilizing the immune cells to destroy the virus cells. The infected person with a strong immunity generates an appropriate level of immune reaction against 2019-nCoV. The development of the disease could be controlled by mobilizing the body’s immune cells (lymphocytes, white blood cells, neutrophils, etc.) against 2019-nCoV. TCM is broadly adopted in the treatment of COVID-19 because it improves and maintains the immune system. However, for those with defective immunity, they are likely to exert overactive immune responses, which causes other healthy cells to be accidentally injured. During the operation of the immune system, it is difficult for the body to accurately recognize the disguised virus cells, resulting in indiscriminate attacks on the cells and thus, causing a storm of immunity and inflammation, which dramatically worsens the physical condition of the infected body.

As an external intervention, TCM and other medical treatments in Western biomedicine that tend to share a common basis with the dynamic disease paradigm (Dong and Zhang 2001; Herfel et al. 2011) play a non-negligible role in the treatment of COVID-19 by not intending to directly cancel the surfacing symptoms of the disease from the beginning, but complying with fundamental rules of the human body and adopting conservative measures to allow the infected person to improve his/her immunity over time. Hence, the inclination of an anaphylactic reaction is weakened. During the process of treatment along a relatively-long course, the infected person with strong immunity may win the fight between the COVID-19 and his/her immune system. In other words, through the proper supplementary treatment compatible with the complex dynamics orientation, the infected person may get to recover.

### Dynamics uncovered from triadic closure to quadrangular cycle

When someone is infected with 2019-nCoV, causation appears showing the 2019-nCoV causes symptoms such as fever and dry cough (2019-nCoV intensity→symptoms of disease). TCM holds that, in essence, the symptoms of body surface indicate the disorders of internal organs (symptoms of disease→organ changes). Fever and inflammation indicate that the infected body is trying to suppress the virus. These symptoms are beneficial to avoid the probable occurrence of anaphylaxis and allow the infected body to gain time to adjust, resulting in an internal adaptation needed.^2^ Meanwhile, the 2019-nCoV damages the organs. Particularly when the attack of coronavirus is too severe, or if it dexterously disguises itself to make the immune organ tolerate it similar to “self-antigens,” the infected body will suffer from unexpected organ changes (2019-nCoV intensity > −−  organ changes). When infected by the coronavirus, the immune organs would produce antibodies. However, when antibodies bind to antigens, the histamines released by the basophils and mast cells may lead to the occurrence of immediate hypersensitivity.

**Fig. 4:**
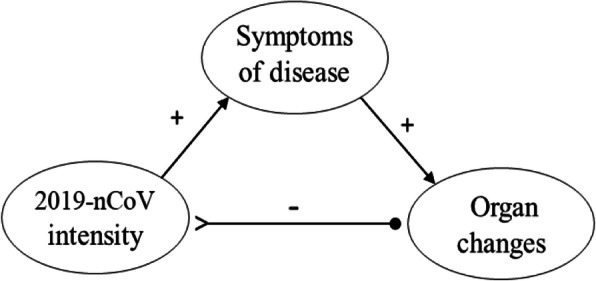
A *PPN* triad of transitive closure

The triadic closure (as shown in Fig. 4) constituted by 2019-nCoV intensity, symptoms of the disease, and organ changes contain two positive links and one negative link, thus forming a triadic structure of *PPN*. The 2019-nCoV intensity, as the reactant, makes the organs lesioned to some degree through the conjunctive effect of direct negative influence and indirect positive influence via the mediation of symptoms of the disease. Since there are two arcs incoming to the resultant (organ changes), the *PPN* is an imbalanced triad of transitive closure, according to FSBH.

The immune system is the body’s intrinsic mechanism that defends against foreign pathogenic microorganisms, including bacteria, viruses, and tuberculosis. Upon the recognition of the invaded virus, the body activates its immune reaction to fight against the virus. But due to its “disguise,” it takes time for the body to judge the coronavirus as antigens of harm. Thus, the infected body spontaneously instructs the immune system to mobilize the cells in various immune organs to react (organ changes→immune reaction), leading several immune cells and free radicals to attack the virus for seemingly providing defense for the body. That is, the immune system generates immune reaction for constraining the 2019-nCoV (immune reaction > −− 2019-nCoV intensity). The intensity of 2019-nCoV is actually a force-countering process between 2019-nCoV as an external force that attacks the body and the immune system as an internal force that defends the body. The net outcome depends on the result of the contest between the two forces. If the immune reaction is proper and effective, the influence of coronavirus is minor. Or else, the infected body suffers.

**Fig. 5:**
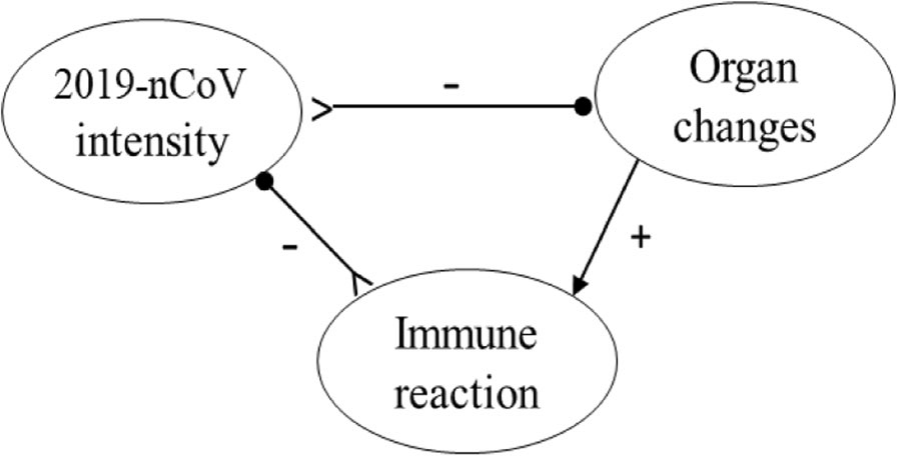
An *NPN* triad of cyclic closure

For the infected person with strong immunity, the change of immune organs is in a mild course toward the gradual and eventual production of antibodies corresponding to the antigens. Thus, the directed negative link from the intensity of the 2019-nCoV to organ changes (2019-nCoV intensity > −−  organ changes) is weakened when the body’s endogenous immunity attacks the virus. Thus, the triadic cycle constituted by 2019-nCoV intensity, organ changes, and immune reaction contains two negative and one positive links (organ changes→immune reaction > −−  2019-nCoV intensity > −−  organ changes), i.e., *NPN*, which indicates the formation of a cyclic loop for attacking the attacker (Fig. 5). According to system dynamics, it is a cyclic structure characterized by an imbalance derived from the mechanism of reinforcing feedback-loop.

As an external intervention, TCM plays a vital role in the treatment of COVID-19 because it does not act immediately to eliminate the symptoms. It pays utmost attention to locating the factor that improves body immunity, which strengthens the power of the immune system in its fight against the coronavirus. TCM terms immunity as vital energy and holds that the body could control the development of disease and recover when this vital energy is sufficient. When the vital energy is deficient, the disease is likely to bring about pathological changes to organs (Dong and Zhang 2001). This means that the key to the defense and treatment of COVID-19 is the maintenance of vital energy.

**Fig. 6:**
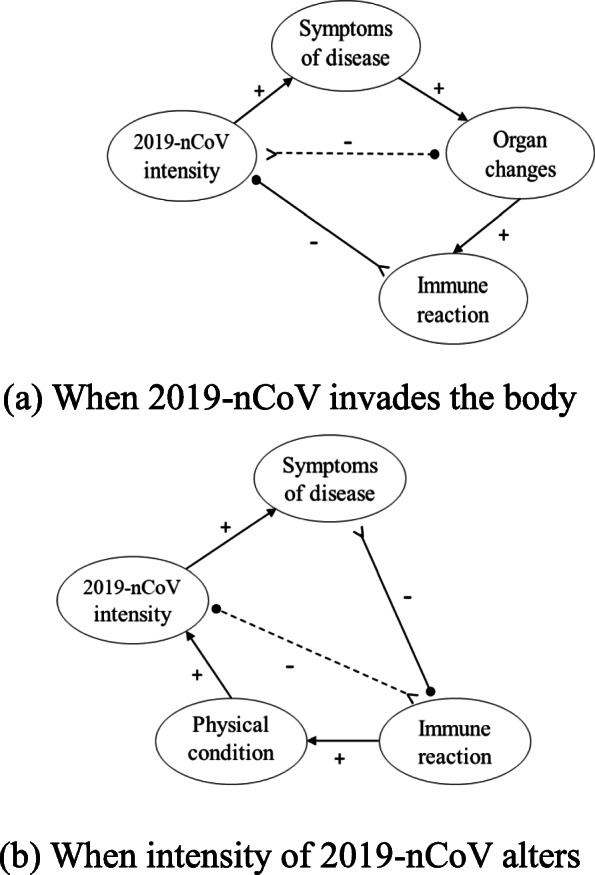
The two quadrangular cycles in succession

According to the above analyses, a sequential positive interlink exists along the chain of 2019-nCoV intensity, symptoms of the disease, organ changes, and immune reaction. The chained interlink indicates the formation of a larger structure based upon the conjunction of four interactive elements. In the quadrangular cycle, observed when the two different triads are placed consecutively in an integrated diagram (as shown in Fig. 6a), the 2019-nCoV intensity, as the reactant, influences the infected body to some degree of change. The result is a change in the state of the body’s organs, which is completed by the mediator (symptoms of disease). The net outcome is the aggregated result of indirect enabling force and direct constraining force from the same reactant (2019-nCoV intensity). Due to the suppression of constraining force from the reactant, the resultant (organ changes) is generated slowly. Then the resultant, as a self-adaptive agent, generates its catalyst (immune reaction) to put constraints on the reactant, resulting in the decrease of constraining force received by the resultant. Thus, the generation process of resultant is accelerated. The quadrangular cycle constituted by reactant, mediator, resultant, catalyst contains three positive links and one negative link, thus forming a balancing feedback-loop of *PPPN*. This cycle is a balanced structure that promises the stable and orderly generation of the targeted resultant.

The infected persons with strong immunity would generate moderate immune reactions and exert constraining force on the intensity of the 2019-nCoV, and gradually return to health. However, infected persons with deficient immunity would generate overactive immune responses such as anaphylactic shock. This activates the next quadrangular cycle. After the first quadrangular cycle is complete, the catalyst (immune reaction) that has completed its role may transform itself into the reactant of the continuous process in the quadrangular cycle (as shown in Fig. 6b). Thus it activates the generation of the next resultant (2019-nCoV intensity). That is, the immune reaction of the infected person as a reactant affects the intensity of 2019-nCoV under change via the mediation of physical condition. A body with strong immunity can reasonably and orderly mobilize the body’s immune cells according to the infection degree of 2019-nCoV to destroy the coronavirus and help the infected body recover gradually. However, when the coronavirus infects people with low immunity, the immune cells are prone to attacking cells, especially healthy cells making immune cells fight the infected organs. This is found to be the root cause of anaphylaxis, which includes inflammation, swelling, redness, fever, tingling, itching, anaphylactic shock, and organ failure.

Anaphylaxis destroys the body’s normal and abnormal cells, making the infected person weaker. The poor state of physical condition, in turn, makes the 2019-nCoV more virulent (physical condition→2019-nCoV intensity), and the increasing degree of infection of coronavirus causes more symptoms of the disease (2019-nCoV intensity→symptoms of disease). Symptoms such as fever indicate that the body is suppressing the coronavirus, and consuming drugs in haste to eliminate the symptoms may make the immune reaction more acute and aggressive. However, allowing the fever to persist for a while but keeping it within an acceptable limit may help the infected person avoid the subsequent inflammation (symptoms of disease > −−  immune reaction). Under the current medical condition that there is no effective anti-viral drug against 2019-nCoV, the infected person demands the body to produce a moderate immune response to fight with 2019-nCoV (immunity reaction > −−  2019-nCoV intensity). TCM, good at improving the immunity, could prevent the progression of the disease and protect the organs from complications, thus enhancing the chance of activating the body’s moderate immune response, helping the recovery of an infected person.

This paired process of cyclic closure brings out a state change of the prior reactant. The second quadrangular cycle, constituted by the immune reaction (reactant), physical condition (mediator), 2019-nCoV intensity (resultant), and symptoms of the disease (catalyst), is isomorphic to the first cycle.

Sequentially, with the closure of attacking the attacker (Chase 1980), the third quadrangular cycle is ready to be activated if the catalyst of the second quadrangular cycle (symptoms of disease) transforms its role into the succeeding reactant to generate the change of state of the forthcoming resultant (immunity reaction), indicating another iteration of the quadrangular cycle. Hence, with the procession of regenerating the reactant, there appears a series of phase transitions.

### From quadrangular cycle to five-nodal cycle (pentagon)

**Fig. 7:**
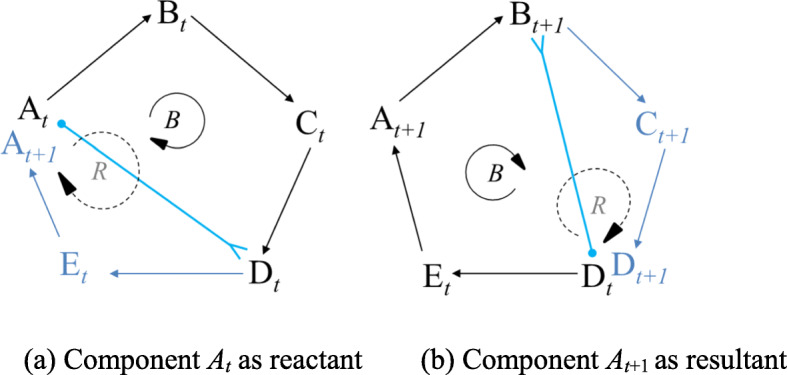
Component *A* plays the role of reactant (*A*_*t*_) and resultant (*A*_*t* + 1_) in turn. *Notes.* B: Balancing mechanism; R: Self-reinforcing mechanism

Simply put, we represent the above five components (2019-nCoV intensity, symptoms of disease, organ changes, immune reaction, and physical condition) by *ABCDE* as the generic codes for the focal five-nodal network/system. Putting the first and second quadrangular cycles into integration, we observe a structure in the shape of a pentagon. For illustration, Component *A* (2019-nCoV intensity), the reactant in the first cycle, transforms into the resultant in the second quadrangular cycle. In the pentagon-shaped structure, shown in Fig. 7a, both the reactant and the resultant are *A*, differing in state. Here, *A*_*t*_, and *A*_*t* + 1_ indicate the changes of component *A*.

In the first quadrangular cycle from *A*_*t*_ to *D*_*t*_, which is governed by the inherent balancing feedback mechanism, the resultant *C*_*t*_ is generated stably owing to the energy transference from *A*_*t*_ as reactant, and component *D*_*t*_ catalyzes the process. Similarly, in the successive quadrangular cycle from *D*_*t*_ to *B*_*t* + 1_, governed by the balancing feedback mechanism, *A*_*t* + 1_ is generated stably given the energy stored in component *D*_*t*_ that is (re-)used as a reactant, and component *B*_*t* + 1_ serves as a catalyst in the second generation process of cyclic closure as shown in Fig. 7b. Following that is the third quadrangular cycle from *B*_*t* + 1_ to *E*_*t* + 1_, and the like.

We thus infer that every component of the five-phase system firstly acts as a reactant to transfer the energy for reproducing its constrainee (targets of the current generation process). Then it gets a payoff as resultant in the succeeding cycle to absorb the energy transferred from the specific reactant. This acts as a catalyst in the prior cycle; then, the reactant at the moment will get repaid in the forthcoming cycle. While each of the five components within a five-phase system has transformed from reactant to resultant, the entire system will finish a complete cycle at the macroscopic level. And because each component taking the turn as resultant will achieve its state change under the mechanism of balancing feedback in each quadrangular cycle, the whole cyclic process of the five-phase system presents itself as a spiral that operates under the mechanism of self-reinforcing feedback.

Therefore, we conclude that the five-phase system constituted by five positive and five negative links is a sound *simplex* model to portray a spiraling process of substantive generation through five phases of energy transference. This dynamic process is characterized by circular causality; that is, the reactant (cause) of the current cycle turns into the resultant (effect) of the next cycle.

### From the pentagon to five-phase system

After the completion of one quadrangular cycle, which implies that the process of generating a specific resultant is complete, the catalyst turns into a reactant in the next quadrangular cycle. This activates another quadrangular cycle to reproduce the resultant which was the previous reactant. If we express the first quadrangular cycle as *AB****C****D*, then the second quadrangular cycle could be expressed as *DE****A****B*. Iteratively, the subsequent quadrangular cycles could be expressed as *BC****D****E*, *EA****B****C*, *CD****E****A*, *AB****C****D* (the resultant is shown in bold). From this series, we observe that the sixth and the first quadrangular cycles are isomorphic. This indicates that after five quadrangular cycles, the whole system achieves an overall development of five-phase transitions in one spiraling period. The resultant of each phase-transition in a sequence is *C*, *A*, *D*, *B*, *E*, then returns to *C*, which implies that the late-coming *C* replaces the previous one. Hence, the whole process is a type of spiraling development of the five-phase system.

**Fig. 8:**
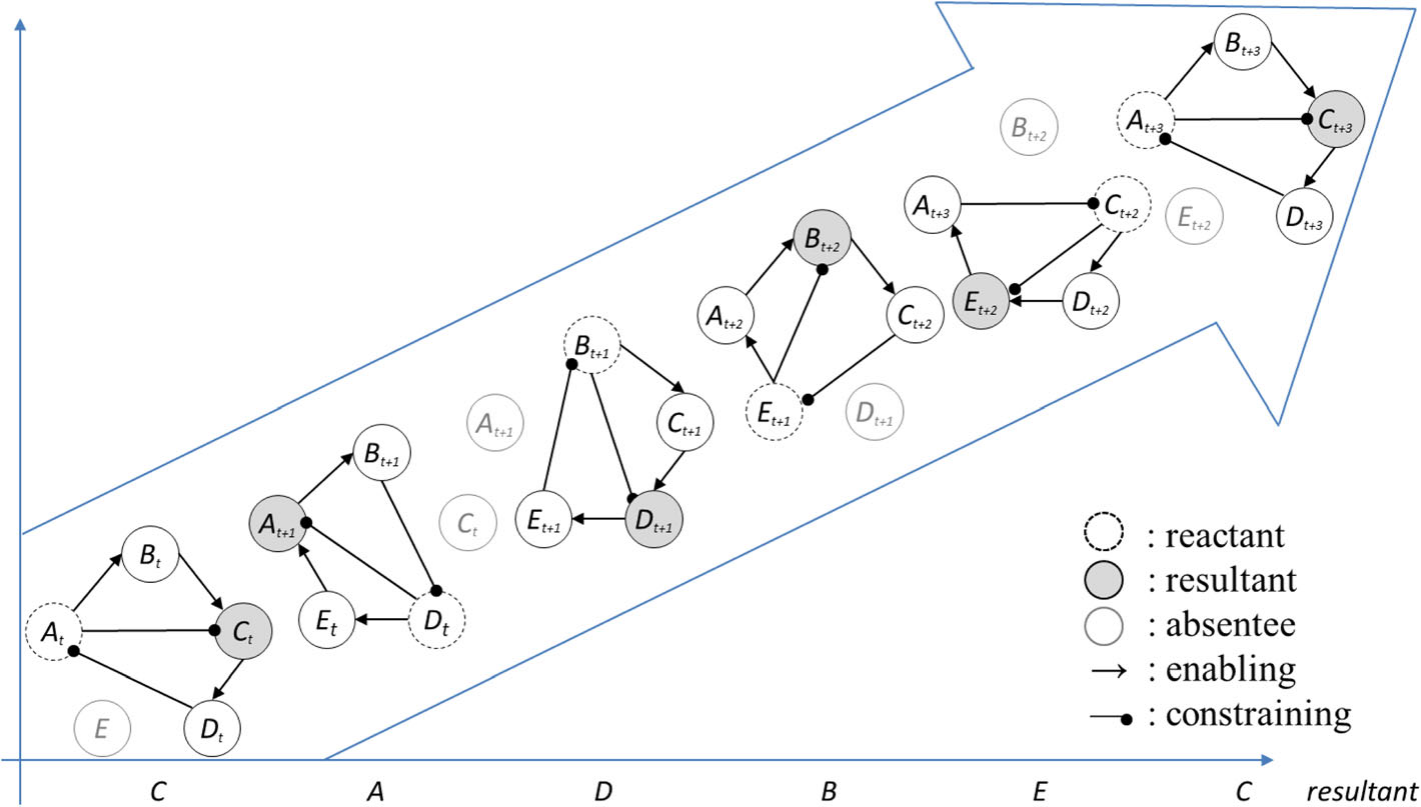
The dynamic upward trajectory of five-phase system

Overlaying the first-round five quadrangular cycles, we could observe the statically-pictured structure of the “five-element system” (as shown in Fig. 3). Herein, we can see the inappropriateness for the sloppy displacement of a spiral with a seemingly repetitious cycle, as portrayed by the frequently adopted but misleading translation of *wuxing* into “five elements.” Factually, if we expand this compressed five-element model along the temporal dimension, we could get a dynamic trajectory of continual spiraling shown in Fig. 8. And every quadrangular cycle in the spiral is qualified as a balanced intermediate form of the five-phase system. It is a stable carrier supporting the orderly generation of various resultants.

Notably, while acting as a reactant in the first quadrangular cycle, component *A* gets its energy gradually consumed. However, in the second quadrangular cycle, the same component turns into a resultant, and thus gradually gains the supplement of formerly-consumed energy. A similar situation occurs with other components, such as *D*, *B*, *E*, and *C*. While the reactant of the previous cycle becomes the resultant of the current cycle, the energy consumption and replenishment of each component achieves dynamic balance.

## Discussion and conclusion

Considering the development of COVID-19 as a metaphor of phase transitions of complex systems, the research attempts to reveal the mechanisms of the influence of 2019-nCoV on the infected person. This is an analogy to the environmental turbulence on the focal organization from the holistic, process-oriented perspective of the five-phase system that is configured as a pentagon with the interweaving of five positive links and five negative links. The observed interweaving positively and negatively directed links among the five elements (2019-nCoV intensity, symptoms of disease, organ changes, immune reaction, and physical condition) constitute a five-phase system. This unveils the causal relationships and dynamic processes of the occurrence and development of COVID-19 within a human body and provides metaphorical insights on the process study of the organizations and other dynamic complexes through the methodology of domain-interaction inquiry (Örtenblad et al. 2016).

The research has reviewed different claims between system dynamics and structural balance theory. It combines the two disciplines to analyze the microstructure of the five-phase system. Triadic closure is not only a salient issue of network analysis but also the basic unit of the five-phase system, which contains transitive triads and cyclic triads. The transitive triad, constituted by two positive links and one incoming negative links, i.e., *PPN,* is imbalanced according to FSBH. This holds that the triad with double receive is balanced if the signs of interlinks are both positive and imbalanced if the two same-directed relations are signed differently. In comparison, the triad constituted by one positive link and two negative links is a loop of cyclic closure, in which the links are unidirectional. Therefore, it is a structure applicable to system dynamics. System dynamics proposes that a cycle with an even number of negative links is imbalanced due to the reinforcing feedback mechanism. In contrast, a cycle with an odd number of negative links is balanced due to the balancing feedback mechanism. We thus infer that the cyclic triad *NPN* in the five-phase system is imbalanced because two negative links imply the cycle is driven by reinforcing feedback mechanism.

Integrating the two kinds of triads, one transitive but imbalanced, and the other cyclic but imbalanced, we observe a quadrangular cycle of balance, which is constituted by three positive and one negative links. The constructed quadrangular cycle in the shape of *PPPN* contains four components acting respectively as reactant, mediator, resultant, and catalyst. It is a balanced cyclic structure operating for the generation of a specified resultant. Owing to the balancing feedback mechanism embedded in the *PPPN* structure, the generation process of the resultant is stable. And when the catalyst of the previous quadrangular cycle turns into the reactant, the next quadrangular cycle is activated. After five quadrangular cycles, the sixth cycle is isomorphic to the first one. This indicates the iteration of cyclic closure. This means that the whole five-phase system achieves updates and substantive change. The replacement and alternation of successive quadrangular cycles promote the spiraling development of the five-phase system. The research uncovers microstructures and the upward trajectory of the self-organizing five-phase system.

### Theoretical contributions

The analogical reasoning promotes the researchers exploiting the metaphor by applying a simple, more contained case to illuminate a focal aspect of the observed phenomena (Mantere and Ketokivi 2013; Morgan 2006). The process theory using the analogy of the development of the COVID-19 enlightens the formation of an emergent pattern of five-stage phase transitions of the focal systems. This research constructs a model of the five-phase system via the domains-interaction way of metaphor inquiry that goes beyond the simple comparison between existing attributes and contributes to theory elaboration for the management of organizations as complex systems.

Firstly, the research contributes to the cross-disciplinary merger of structural balance theory and system dynamics and promotes the academic dialogue between the Western process study and Chinese *yin* and *yang* and five-phase notions. In the Western disciplines of organization and management, the inquiry on changes and evolutions is frequent and mostly oriented toward line-like (i.e., linear) pattern of causation. This results in the dominance of a steady-state or clock-time perspective (Helin et al. 2014; Reinecke and Ansari 2016) and correlation linking logic that stops at identifying correlations with single elements (Kouamé and Langley 2018). Among the exceptions, there exist different claims between structural balance theory and system dynamics, with the former emphasizes the sign of links. In contrast, the latter explores the closed path of unidirectional links. Our comparative and integrative analysis of transitive closure and cyclic closure in the same five-phase system helps to enhance the synergy of structural balance theory and system dynamics. The two kinds of triads are both not balanced. Still, based on them, a series of quadrangular cycles are sequentially constituted that are balanced under the cyclic closure structured by three positive links and one negative link. The embedded balancing feedback mechanism warrants the stability underpinning this intermediate form. The dynamic, holistic picture of the five-nodal system is congruent with the underlying logic of system dynamics, which extracts the balancing and reinforcing feedback loops. It is also coincident with the Chinese wisdom of the five-phase system.

Secondly, the research expands the understanding of system nonlinearity by visualizing and deepening the meaning of circular causality. The reactant (cause) of the current quadrangular cycle turns into the resultant (effect) of the next quadrangular cycle. Furthermore, during the process of role transformation, the energy of the reactant consumed in the previous cycle is replenished. Additionally, the resultant (effect) of the first quadrangular cycle becomes the reactant (cause) of the fifth quadrangular cycle, reflecting causal recursiveness. On the other hand, the research reveals the coexistence of positive and negative relationships and the influence of the dominant relationship on the nature of the emergent structure. The balancing feedback mechanism embedded in the quadrangular cycle and reinforcing feedback mechanism in the pentagon mutually interact, driving the system into the trajectory of spiraling development. The orderly and regular combination of positive links and negative links leads to periodic cycling through the five phases, reflecting the essence of the five-nodal system in the midst of the interwoven signed and directed relations.

Thirdly, the research enriches the dynamic connotation of the five-phase system through the connections among five processes of phase transition. That is, beyond the static connection between components of the five-nodal system, the research emphasizes on the spiral dynamics of the system evolving in a period of five phases, reflecting that the reality is a process devoid of any solid, substantial objects that can be relied on to stay the same (Zhang 2017). Through a period of the spiral, each node gets updated upon the completion of every phase of state change, and, in the conjunction of five-phase changes, the whole system achieves homeostasis and sustainable development. Based on the identification and differentiation of two types of triads as microstructures and the successive formation of quadrangular cycles, this research uncovers the intermediate form of an evolving five-phase system. While the reinforcing feedback mechanism governs the operation of the whole system with five components, its intermediate form is found as stable quadrangular cycles with embedded balancing feedback mechanisms. As the meso-level structure of the five-phase system, the quadrangular cycles are required to manage the complexity of the five-phase system (Simon 1962). They are also the underlying microstructure supporting the spiraling development of complex system operating as a five-phase system.

Fourthly, the microstructures and dynamic processes of the five-phase system embody and visualize the characteristics of pragmatism philosophy as follows. First, consistent with the pragmatist viewpoints focusing on process and stressing the endogeneity of change, the abducted evolutionary development of the five-phase system is in an ongoing flux, and the process of self-becoming (Farjoun et al. 2015). As shown in Fig. 8, the structures and processes are presented as interrelated pairs of (self-)organization and processual aspects, which are supported by or anchored in structural aspects. Second, the quadrangular cycles and their alternation underline the dynamic relations among interactive components, instead of atomistic entities. They, thus, actualize and strengthen the philosophical assertion on the flux and flow of wholeness as a process (Cooper 2014). Third, the transformation of reactant and resultant and the self-renewal of each node reflect the recursiveness, one of the main principles in pragmatism, which stresses the iterative and cumulative processes that connect back to themselves (Simpson 2009). Finally, the contradictory and complementary coexistence of positive and negative links and the mediator between reactant and resultant are correspondent with the anti-dualism of pragmatism, which regard opposites recursively related to each other.

### Implications and future directions

Regarding practical implications, the development process is a continuous flow. The identification of causal relationships among elements and the sign and direction of relations could help to capture the essence of the problem and help policymakers predict the system evolution and possible results of policy intervention. Negative links are not necessarily unacceptable (Marineau et al. 2016). Managers should be tolerant of the confusion caused by the interweaving of positive and negative relations. Besides, managers should not only focus on reinforcing loop, but also need to place a limit on the entire system to avoid uncontrolled growth or unexpected decline. The target of intervention is the establishment of a natural negative feedback mechanism, which could react with imbalances generated by the reinforcing loop. The harmony achieved through *yin–yang* balance (Li 2012, 2014, 2016) is indispensable to sustainable development. Managers should be aware that underlying micro- and meso-level structures support dynamic and complex processes, and careful analysis of the microstructures can provide valuable references for effective intervention.

Although the research portrays and extracts the microstructures and dynamic processes of the five-phase system through an analogy, it faces the inherent limitation when adopting the metaphorical methodology. Considering the contextual difference between the development of COVID-19 and the systematic changes of real business organizations, a cross-contextual comparison is required to deepen the understanding of the particular context for “making present” of a specific kind of being in the development process^3^ at the specific point in time. Furthermore, by adopting a metaphorical inquiry, this research lacks empirical insights from qualitative and/or quantitative dimensions. Future researches could try the multi-case study using the replication or comparison logic and, perhaps, temporal qualitative comparative analysis (tQCA) to analyze the sequentially and dialectically ordered configurations related to five-phase systems. Besides, the five-phase system contains novel insights into the dynamic process of complex systems from the Eastern-originated holistic and process-oriented view. Proposing an academic dialogue between the five-phase system and Western process theory is also one of the future research directions.

## Data Availability

The data and material of the study are from public information on the Internet.

## Abbreviations

COVID-19: Disease caused by the coronavirus
2019-nCoV: Novel coronavirus
TCM: Traditional Chinese medicine
FSBH: Fundamental structural balance hypothesis

## Footnotes

1 The notion of “simplex” was borrowed from Jeffrey Kluger’s book *Simplexity*: *The Simple Rules of a Complex World* (Klugers 2007)*.* The definition of simplex is similar to that of Cunha and Rego (2010) defining simplexity as the paradoxical notion of a duality made of simplicity and complexity.

2 Needham (1954), the eminent expert of history science, says: “the disclosure of the secret for the necessary connection between the reactions of the human body surface and the changes in the internal organs is the great discovery in terms of physiology in Medieval China.”

3 Wang et al. (2018) carried out a longitudinal comparative case study that takes Haier and IBM as cases to explore the enabling & constraining and metaplasia & inhibitive generation mechanisms in the firms’ process of organizational change. The study connects the context, process and content of organizational change and identifies a close-looped process of organizational change which is composed of such five elements: contextualization, mindset, mode, system and humanity. It is an exploratory study on the endogenous dynamics brought about by either similar or contradicting elements of change contents that are subjected to the dual effects of enabling and constraining. Thus, they provide a preliminary processual explanation on the organizational change phenomena in the real business world.
